# Normalization of Oxygen Levels Induces a Metabolic Reprogramming in Livers Exposed to Intermittent Hypoxia Mimicking Obstructive Sleep Apnea

**DOI:** 10.3390/antiox14080971

**Published:** 2025-08-07

**Authors:** Miguel Á. Hernández-García, Beatriz Aldave-Orzáiz, Carlos Ernesto Fernández-García, Esther Fuertes-Yebra, Esther Rey, Ángela Berlana, Ramón Farré, Carmelo García-Monzón, Isaac Almendros, Pedro Landete, Águeda González-Rodríguez

**Affiliations:** 1 Research Unit, Hospital Universitario Santa Cristina, Instituto de Investigación Sanitaria Hospital Universitario de La Princesa, 28006 Madrid, Spain; miguelangel.hernandezg@estudiante.uam.es (M.Á.H.-G.); cernesto.fernandez.garcia@gmail.com (C.E.F.-G.); esther.fuertes@salud.madrid.org (E.F.-Y.); erfernandez@salud.madrid.org (E.R.); angela.berlana.22@gmail.com (Á.B.); garciamonzon@hotmail.com (C.G.-M.); 2 Respiratory Medicine Department, Hospital Universitario de La Princesa, Instituto de Investigación Sanitaria Princesa Hospital Universitario de La Princesa, 28006 Madrid, Spain; beatrizaldave@gmail.com (B.A.-O.); landete.pedro@gmail.com (P.L.); 3 Instituto de Investigaciones Biomédicas Sols-Morreale (IIBM), Consejo Superior de Investigaciones Científicas—Universidad Autónoma de Madrid, 28029 Madrid, Spain; 4 Unitat de Biofísica i Bioenginyeria, Facultat de Medicina i Ciències de la Salut, Universitat de Barcelona, 08036 Barcelona, Spain; rfarre@ub.edu (R.F.); isaac.almendros@ub.edu (I.A.); 5 Centro de Investigación Biomédica en Red de Enfermedades Respiratorias (CIBERES), 28029 Madrid, Spain; 6 Instituto d’Investigacions Biomèdiques August Pi I Sunyer (IDIBAPS), 08036 Barcelona, Spain; 7 Centro de Investigación Biomédica en Red de Diabetes y Enfermedades Metabólicas Asociadas (CIBERDEM), 28029 Madrid, Spain

**Keywords:** obstructive sleep apnea, CPAP, MASLD, liver steatosis, intermittent hypoxia, steatosis, lipid metabolism

## Abstract

Obstructive sleep apnea (OSA), characterized by intermittent hypoxia (IH), is strongly associated with metabolic syndrome and metabolic dysfunction-associated steatotic liver disease (MASLD). IH exacerbates MASLD progression through oxidative stress, inflammation, and lipid accumulation. This study aims to investigate the impact of oxygen normalization on metabolic dysfunction in OSA patients using continuous positive airway pressure (CPAP) therapy, and in mice exposed to IH followed by a reoxygenation period. In the clinical study, 76 participants (44 OSA patients and 32 controls) were analyzed. OSA patients had higher insulin resistance, triglycerides, very low density lipoprotein (VLDL) content, and liver enzyme levels, along with a higher prevalence of liver steatosis. After 18 months of CPAP therapy, OSA patients showed significant improvements in insulin resistance, lipid profiles (total cholesterol and VLDL), liver function markers (AST and albumin), and steatosis risk scores (Fatty Liver Index and OWLiver test). In the experimental study, IH induced hepatic lipid accumulation, oxidative stress, and inflammation, and reoxygenation reversed these deleterious effects in mice. At the molecular level, IH downregulated fatty acid oxidation (FAO)-related genes, thus impairing the FAO process. Reoxygenation maintained elevated levels of lipogenic genes but restored FAO gene expression and activity, suggesting enhanced lipid clearance despite ongoing lipogenesis. Indeed, serum β hydroxybutyrate, a key marker of hepatic FAO in patients, was impaired in OSA patients but normalized after CPAP therapy, supporting improved FAO function. CPAP therapy improves lipid profiles, liver function, and MASLD progression in OSA patients. Experimental findings highlight the therapeutic potential of oxygen normalization in reversing IH-induced liver damage by FAO pathway restoration, indicating a metabolic reprogramming in the liver.

## 1. Introduction

Obstructive sleep apnea (OSA) is a highly prevalent respiratory disorder characterized by recurrent episodes of partial or complete upper airway obstruction during sleep, leading to intermittent hypoxia (IH) and sleep fragmentation. Recent research estimated that the prevalence of OSA ranges from 9% to 38% in the general population, with higher prevalence rates observed in individuals with obesity and other components of metabolic syndrome [1]. The strong association between OSA and metabolic syndrome is underpinned by shared risk factors, including central obesity, insulin resistance, dyslipidemia, hypertension, and circadian-clock disruption [2,3,4]. OSA exacerbates these metabolic disturbances through mechanisms such as IH-induced oxidative stress, systemic inflammation, and autonomic nervous system activation, creating a vicious cycle that perpetuates both conditions [2].

In this regard, OSA is closely related to the pathogenesis of metabolic dysfunction-associated steatotic liver disease (MASLD) [5], formerly known as non-alcoholic fatty liver disease, the hepatic manifestation of metabolic syndrome. This disease is the most common chronic liver disorder worldwide, affecting up to 30% of the global adult population [6]. MASLD encompasses a spectrum, from simple steatosis, characterized by lipid accumulation in more than 5% of hepatocytes, to metabolic dysfunction-associated steatohepatitis, which can progress to fibrosis, cirrhosis, or hepatocellular carcinoma [7,8]. The molecular basis for MASLD progression lies in the disruption of hepatic lipid homeostasis. Pathological lipid accumulation in hepatocytes is due to a dysregulation between four main pathways: the uptake of circulating lipids, de novo lipogenesis (DNL), lipid export in very low density lipoproteins (VLDLs), and free fatty acid (FFA) oxidation (FAO) [9,10]. The latter process is mediated by the mitochondria, peroxisomes, and cytochromes in mammalian cells. Entry of FFA into the mitochondria relies on the carnitine palmitoyltransferase (CPT) enzyme system, but as the mitochondria lack the ability to oxidize very long chain FFA, these are preferentially metabolized via peroxisomal β-oxidation. In case of lipid overload, such as in MASLD, ω-oxidation in the cytochromes also contributes to FAO [10]. Peroxisome proliferator-activated receptors (PPARs) are members of the steroid hormone receptor superfamily of ligand-activated transcription factors that play a crucial role in regulating lipid metabolism, with some of their targets including genes involved in DNL (*Fasn*), as well as mitochondrial and peroxisomal β-oxidation [11,12].

Emerging evidence suggests a bidirectional relationship between these conditions, with OSA exacerbating MASLD progression and vice versa, underscoring the need for integrated approaches to management and treatment. The pathophysiological link between OSA and MASLD is multifaceted, involving shared risk factors such as obesity, insulin resistance, and systemic inflammation. Intermittent hypoxia (IH), a hallmark of OSA, plays a pivotal role in this interplay. IH induces oxidative stress, promotes hepatic lipid accumulation, and triggers pro-inflammatory pathways, all of which contribute to the progression of the liver disease [13]. Additionally, OSA-associated sleep fragmentation and chronic sympathetic activation further exacerbate metabolic dysregulation, compounding the risk of hepatic steatosis and progression to steatohepatitis, fibrosis, and cirrhosis [2].

Continuous positive airway pressure (CPAP) therapy is the standard treatment for OSA. By providing constant airflow, CPAP mitigates airway collapse, restores normal oxygenation, and improves sleep quality. The efficacy of CPAP has been demonstrated in numerous studies, highlighting its role in reducing the apnea–hypopnea index (AHI), normalizing sleep architecture, suppressing sleepiness, and improving quality of life in patients with OSA [14].

Taking into account this information, an improvement or even a resolution of the OSA-associated liver damage would be expected after the correction of IH by CPAP use. However, with the scientific evidence available to date, there is still some controversy about the benefit of CPAP at the hepatic level [15].

Due to the lack of treatments for MASLD and its association with OSA, this study aims to elucidate the impact of normalization of oxygen levels (reoxygenation) on MASLD progression. To this end, a clinical cohort of OSA patients undergoing CPAP treatment was analyzed, along with a histological and molecular analysis of the livers from mice exposed to a protocol of IH followed by reoxygenation in order to avoid the confounding factors that exist in patients with OSA. By integrating histological, molecular, and clinical data, this study aims to clarify whether CPAP treatment of OSA could serve as a therapeutic strategy to mitigate MASLD and its complications.

## 2. Materials and Methods

### 2.1. Study Population

This longitudinal study included patients with clinical and polygraphic criteria of OSA who attended the outpatient clinics of the Pneumology Service at Hospital Universitario de La Princesa (HULP, Madrid, Spain) during a 3-month period, and who maintained CPAP treatment for 18 months after the diagnosis of OSA. In parallel, volunteers who had sleep polygraphy within normality were included in this study and considered to be controls (subjects with normal lung function parameters, NLPs).

This study was performed in agreement with the Declaration of Helsinki, and with local and national laws. The Clinical Research Ethics Committee of the Institution (Instituto de Investigación Sanitaria del HULP, Madrid, Spain) approved the study procedures (Report Reference: PI/2800-16), and all participants signed informed written consent before inclusion in this study.

### 2.2. Polygraphic Study

All polygraphic studies were performed at night in the patients’ homes, using unattended domiciliary cardiorespiratory polygraphy. The recordings were conducted by the patients’ usual caregivers following standardized procedures. The parameters measured included airflow measurement with an oronasal thermistor and nasal pressure transducer; thoracoabdominal movement, assessed by impedance plethysmography; pulse oximetry; and microphone recording to evaluate snoring, breathing patterns, and movement. A previously validated cardiorespiratory polygraphy device (SOMNOscreenTM Plus, Randersacker, Germany) with DOMINO analysis software 2.8.0 (Domino Data Lab, San Francisco, CA, USA) was used. For interpretation, the recommendations of the American Academy of Sleep Medicine for OSA in adults were followed. The polygraphic records were manually scored, assisted by DOMINO analysis software 2.8.0.

Apnea was defined as the absence of oronasal airflow and absence of signal in the thermistor for more than 10 s, and hypopnea as a decrease in baseline airflow between 30 and 90%, accompanied by a significant desaturation (oxygen saturation drop >3% from baseline). Apnea events were further classified as central or obstructive. Central apnea was defined as the absence of oronasal airflow and thoracoabdominal movements, with no associated body movements. Obstructive apnea was defined as the interruption of airflow in the nose and mouth, associated with thoracic and abdominal movements.

The apnea–hypopnea index (AHI), indicating the number of these events per hour of recording, was used as the diagnostic criterion for OSA severity: AHI < 5 h^−1^, no OSA; AHI 5–14 h^−1^, mild OSA; AHI 15–29 h^−1^, moderate OSA; and AHI ≥ 30 h^−1^, and severe OSA. An AHI ≥ 5 h^−1^ was used as the diagnostic threshold for OSA. Additional polygraphic parameters analyzed included the Oxygen Desaturation Index (ODI), defined as the number of ≥3% desaturations per hour of recording, and the percentage of recording time with oxygen saturation below 90% (Tc90%). Both ODI and Tc90% were considered low when <10 events/hour or <10%, respectively, and high when ≥10 events/hour or ≥10%, respectively.

### 2.3. Inclusion and Exclusion Criteria

Inclusion criteria for OSA patients into the study consisted of presenting an AHI ≥ 5 h^−1^ in the nocturnal polygraph recording. Control patients (NLPs) were those with an AHI < 5 h^−1^. Patients and controls were excluded if they had a diagnosis of asthma or cancer, or any condition with a life expectancy of less than one year, as well as those who did not agree to participate in the study. Also excluded were patients with excessive alcohol intake (≥30 g/day in men and ≥20 g/day in women) or other liver diseases (analytical evidence of iron overload—transferrin saturation index 55%; seropositive for autoantibodies, and/or for hepatitis B virus, hepatitis C virus, or HIV; and/or used potentially hepatotoxic drugs).

### 2.4. Clinical and Laboratory Assessment

Clinical examination was performed on all participants in this study. Various anthropometric variables were collected, and different biochemical and metabolic variables were determined in the NLP subjects and OSA patients’ fasting serum. In addition to this data collection at the time of OSA diagnosis, serum samples and data were collected again after 18 months of CPAP treatment. This data collection allowed for a pre- and post-treatment study of the different variables, thus verifying the effects of CPAP and any possible clinical improvements. This data collection was carried out by clinical staff from the HULP.

Blood-based scores for the non-invasive determination of metabolic dysfunctions, such as HOMA-IR [16], Fatty Liver Index (FLI) [17], Hepamet Fibrosis Score (HFS) [18], and Non-Alcoholic Fatty Liver Disease Fibrosis Score (NAFLD FS) [19], were calculated in the study population.

### 2.5. Serum Metabolomic Analysis

OWLiver™ test is a commercially available assay used to determine serum lipidomic profiling. It was validated to distinguish normal liver (0), simple steatosis (1), and steatohepatitis (2) with excellent diagnostic accuracy [20].

### 2.6. Animal Care and Intermittent Hypoxia Protocol Followed by Reoxygenation

Male and female C57BL/6J mice, 12 weeks old, from Charles River Laboratories (Saint-Germain-Nuelles, France) were exposed to an IH protocol followed by a normoxic period.

(1) IH group: Every minute, mice received air with 5% oxygen for 20 s, followed by 40 s of ambient air, for 6 h a day, 5 days a week, for a total of 8 weeks [21]. Control mice were maintained under normoxic conditions (N) during the 8 weeks.

(2) Reoxygenation (Reox) group: The mice underwent the same IH protocol, followed by a 4-week period of exposure to normoxia. Control mice were maintained under normoxic conditions (C) during the course of 12 weeks.

At the end of the experiment, the mice were anesthetized and sacrificed, and their livers were removed. All experimental procedures were approved by the Ethical Committee of the University of Barcelona (174/18-10268).

### 2.7. Histopathological Evaluation

Liver tissues were fixed in 4% PFA and embedded in paraffin. Sections of 4 µm thickness were obtained using a rotary microtome and stained with hematoxylin–eosin (H&E). The histological sections were blindly examined by an expert pathologist. Histopathological classification followed the system described by Kleiner et al. [22], known as NAFLD Activity Score (NAS), evaluating the degree of steatosis, lobular inflammation, and hepatocellular degeneration.

### 2.8. Assessment of Lipid Accumulation

Liver tissues were fixed and embedded in Tissue-Tek^®^ O.C.T.™ compound (Sakura Finetek Europe, Alphen aan den Rijn, Netherlands). Sections of 10 µm thickness were obtained using a Leica CM1510S cryostat. These sections were stained with Oil Red O (ORO, Sigma-Aldrich, St. Louis, MO, USA) working solution (60% ORO/isopropanol *w*:*v*) and counterstained with hematoxylin (1.09235.0500, PanReac AppliChem, ITW Reagents, Castellar del Vallès, Spain).

### 2.9. IBA1 (Ionized Calcium-Binding Adapter Molecule 1) Immunohistochemistry

Paraffin-embedded liver biopsy sections (5 µm) were deparaffinized overnight at 65 °C and rehydrated through a descending series of ethanol concentrations. Tissue was pretreated using the heat-induced epitope retrieval method. Endogenous peroxidase was blocked with 3% hydrogen peroxide in methanol for 20 min, and samples were submerged in 0.01 mM sodium citrate pH 6 and heated for 15 min. Tissue was permeabilized with 1% Triton-100 in phosphate buffer (PBS) for 7 min, and antigens were unmasked with 0.04% pepsin in 0.1M HCl for 20 min. Tissue was incubated with primary anti-IBA1 antibody 1:1000 (ab5076, Abcam plc, Cambridge, UK) in 10% goat serum overnight in a humid chamber at 4 °C. Then, samples were incubated with the secondary antibody: rabbit anti-goat immunoglobulin diluted 1:100 in PBS for 1 h at room temperature (RT) in a humid chamber. Samples were then incubated with the EnVision™ Flex DAB+ Substrate Chromogen System (GV825, Dako Omnis, Agilent, Santa Clara, CA, USA) at RT. Finally, tissues were counterstained with Harris hematoxylin, dehydrated, and mounted with a coverslip.

### 2.10. 4-HNE (4-Hydroxynonenal) Immunohistochemistry

Paraffin-embedded liver biopsy sections (5 µm) were deparaffinized, rehydrated, and unmasked under the same conditions as immunohistochemistry (IHC) of IBA1. Endogenous peroxidase was blocked with 3% hydrogen peroxide in methanol for 15 min, followed by 15% goat serum in PBS-T (0.1% Triton X-100 in PBS) and 3% bovine serum albumin (BSA) for 1 h at RT. Tissue was incubated with primary anti-4-HNE antibody 1:50 (ab46545, Abcam plc, Cambridge, UK) in PBS-T with 1% BSA overnight in a humid chamber at 4 °C. Then, samples were incubated with secondary antibody: rabbit anti-goat immunoglobulin diluted 1:100 in PBS for 1 h at RT in a humid chamber. Samples were incubated with the EnVision™ Flex DAB+ Substrate Chromogen System (GV825) at RT. Finally, tissues were counterstained with Harris hematoxylin, dehydrated, and mounted with a coverslip.

### 2.11. Analysis of ORO Staining and IHQ Images

Four different lobular areas were analyzed in each sample and photographed using a Nikon 90i optical microscope (Nikon, Tokyo, Japan) and the NIS Elements Imaging Software 3.22 (Nikon Instruments Inc., Melville, NY, USA). Intensity of stain was blindly quantified using ImageJ Biological Image Analysis v1.54p (NIH, Bethesda, MD, USA), and data were normalized with the control mean value and represented in arbitrary units (a.u.).

### 2.12. Fatty Acid Oxidation (FAO) Assay

The activity of fatty acid β-oxidation in mice livers was determined using the Fatty Acid Oxidation Assay Kit (BR00001, Assay Genie, Dublin, Ireland). Absorbance values were obtained using a Dynex Spectra MR Microplate spectrophotometer/computer software SpMR 1.01 (Chantilly, VA, USA) and graphically expressed as FAO activity (O.D. 492 nm).

### 2.13. Quantitative Real-Time PCR (RT-qPCR)

RNA was extracted from liver samples using the TRIzol^®^ reagent (Vitro, Sevilla, Spain). Samples were then reverse-transcribed using the Promega, Inc., Reverse Transcription System (Madison, WI, USA). A BioRad T100™ Thermal Cycler (Hercules, CA, USA) was used to carry out the reverse transcription. Quantitative real-time polymerase chain reaction (RT-qPCR) was performed to assess gene expression using a StepOnePlus™ Real Time PCR System Sequence Detector (Thermo Fisher Scientific, Inc., Waltham, MA, USA). Samples were prepared using a SYBR Green qPCR Kit (Promega Inc., Mumbai, MH, India), and d(N)6 random primers were purchased from Metabion (Planegg, Germany). Primer sequences are detailed in Table 1. Each sample was duplicated, normalized to *36b4* gene expression, and graphically expressed as fold change.

### 2.14. β-Hydroxybutyrate Assay Kit Assay

β-hydroxybutyrate metabolite was determined in plasma samples from NLP subjects and OSA patients using a colorimetric assay (18634911, beta-Hydroxybutyrate Assay Kit, Novus Biologicals™, Centennial, CO, USA). Absorbance values were obtained using a CLARIOstar^Plus^ BMG Labtech (Ortenberg, Germany) and graphically expressed as mmol/L.

### 2.15. Statistical Analysis

Regarding the statistical analysis, the Kolmogorov-Smirnov test was applied to evaluate the fit of the variables to a normal distribution. For the clinical study, the data were presented as mean ± standard deviation (SD) and compared using a paired *t*-test for variables that followed a normal distribution or the Wilcoxon test for non-parametric distribution, for continuous variables pre- vs. post-treatment. An unpaired *t*-test or Mann-Whitney test was performed, respectively, for continuous variables in control and pre-treatment analysis. For categorical variables, the chi-square test or Fisher’s exact test was used as appropriate. For paired categorical data, McNemar’s test was applied. For animal experiments, sample size was chosen based on similar previous studies of our group and on the basis of the literature documentation of similar well-characterized experiments [23,24]. The number of mice were used to achieve statistical significance following 3R rules on animal experimentation. In the experimental study, the data were presented as mean ± standard error of the mean (SEM) and compared using an unpaired *t*-test or Mann-Whitney test. The level of statistical significance was set at *p* < 0.05. All calculations were performed using the IBM SPSS Statistics 21.0 statistical software (SPSS Inc., IBM, Armonk, NY, USA) and GraphPad Prism 9.4.1 software (GraphPad Software Inc., San Diego, CA, USA).

## 3. Results

### 3.1. Characteristics of the Study Population and Prevalence of Metabolic Disorders

This study included 76 patients (44 with OSA and 32 controls). Multiple anthropometric, biochemical, and metabolic variables were recorded (Table 2).

Overall, OSA patients were significantly older than the control population (*p* = 0.0174), and men were predominant in the OSA group, while most individuals in the NLP group were women (*p* = 0.0079). No significant differences were found in BMI. Considering the pulmonary function parameters, as expected, patients with OSA had a significantly higher result for the AHI (*p* < 0.0001).

**Figure 1:**
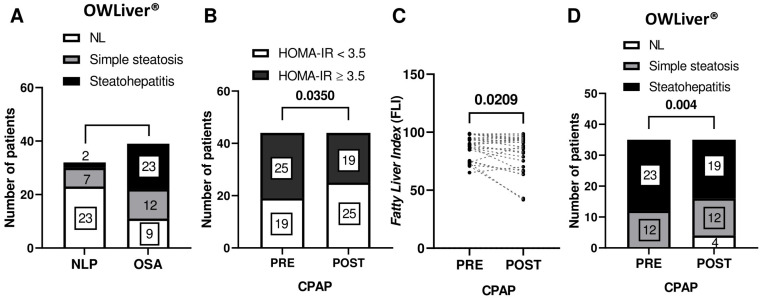
CPAP therapy improved insulin resistance and presence of liver steatosis and steatohepatitis. (**A**) Metabolomic test OWLiver in control (*n* = 32) and OSA group (*n* = 44). (**B**) Insulin resistant state in the study population (*n* = 44) before (PRE) and after (POST) CPAP intervention. (**C**) Fatty Liver Index (FLI) and (**D**) metabolomic test OWLiver in those patients classified as high risk of MASLD, FLI > 60 (*n* = 27) and OWLiver ≥ 1 (*n* = 35), respectively.

In terms of metabolic parameters, the degree of insulin resistance assessed by the HOMA-IR index significantly differed between groups (*p* = 0.0120), being higher in OSA patients. They also had significantly higher levels of triglycerides and VLDL (*p* = 0.0399 and *p* = 0.0339), as well as ALT and GGT liver enzymes (*p* = 0.0116 and *p* = 0.0240). Indeed, when comparing control subjects with OSA patients, Fatty Liver Index (FLI), a simple tool to predict the risk of hepatic steatosis [17], was significantly higher among the OSA population (*p* = 0.0042). Using the OWLiver™ lipidomic test for the non-invasive diagnosis of MASLD, the prevalence of both simple steatosis and steatohepatitis was significantly higher in patients with OSA (28.20% and 43.59%, respectively) than in the NLP group (21.87% and 6.25%, respectively; Figure 1A), indicating that OSA patients were at higher risk of presenting MASLD.

Regarding the effects of CPAP treatment (Table 3), OSA patients before treatment presented an AHI of 41.11 ± 19.71 h^−1^, a diagnosis consistent with severe OSA (30 ≤ AHI < 50 h^−1^), compared to the AHI achieved after 18 months of active treatment, 7.12 ± 15.73 h^−1^, showing values almost within normality. No significant difference was found in BMI after CPAP treatment.

Also, similar glucose and insulin levels were found before and after treatment. However, it should be noted that 58.61% of OSA patients presented insulin resistance (HOMA-IR ≥ 3.5), and, when analyzing only these patients, significant differences were found when comparing HOMA-IR before and after CPAP treatment (Figure 1B): the number of patients with insulin resistance decreased significantly after CPAP therapy (*p* = 0.0350).

Regarding the lipid profile obtained from the blood analysis, a significant change in total cholesterol and VLDL levels was observed, being lower after CPAP treatment (*p* = 0.0044 and *p* = 0.0304), while no statistically significant differences were found in triglycerides or LDL, although an important reduction was observed.

Different markers of liver function were analyzed, including AST levels, which were significantly lower after CPAP treatment (*p* = 0.0088), and, conversely, albumin levels significantly increased (*p* < 0.0001).

Focusing on patients with a high probability of presenting MASLD, those classified as high risk according to a FLI value equal to or higher than 60, as the optimal cutoff point to identify fatty liver (FLI > 60), and OWLiver equal to or higher than 1 (OWLiver ≥ 1), improved their outcomes after CPAP therapy (Figure 1C,D).

### 3.2. Normalization of Oxygen Levels Reverses Lipid Accumulation in Livers from Mice Exposed to IH

**Figure 2:**
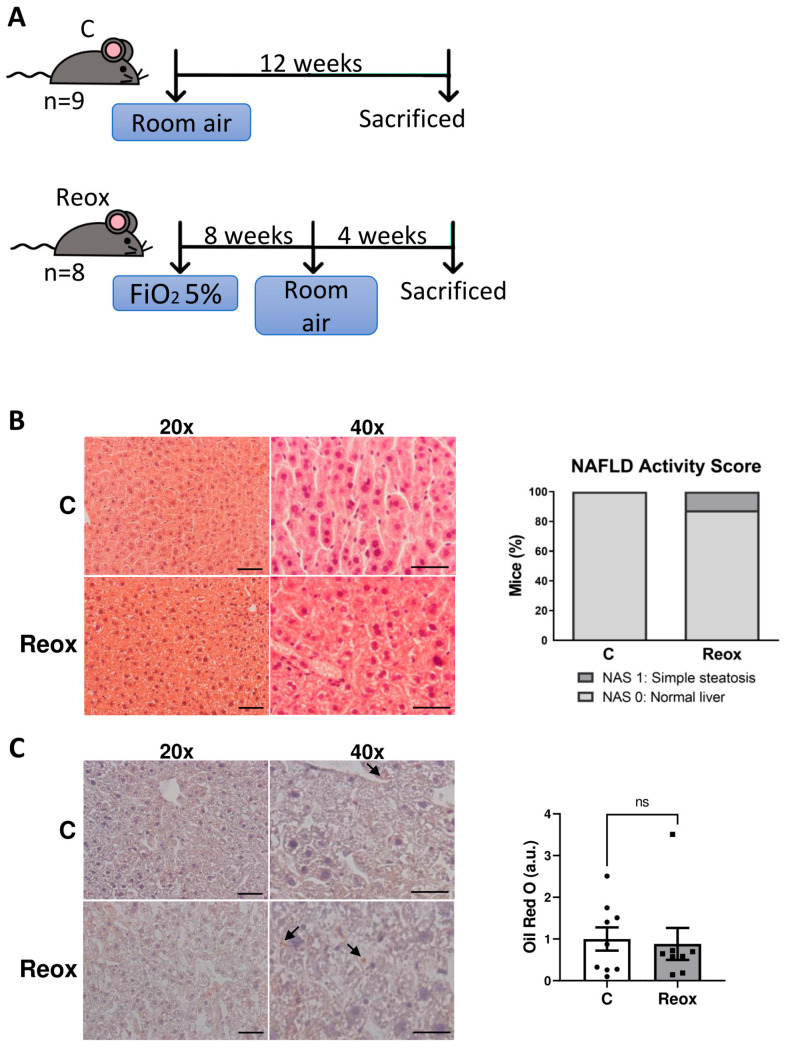
Reoxygenation is sufficient to reverse the hepatic lipid accumulation induced by intermittent hypoxia (IH) in male mice. (**A**) Protocol for exposure to IH intervals followed by reoxygenation (Reox). Reox mice received air with an oxygen fraction (FiO_2_) of 5% in alternating cycles for a total of 8 weeks, followed by 4 weeks of room air exposure. Control mice (C) were only exposed to room air. (**B**,**C**) Representative images at 20× and 40× (scale bar at 100 and 50 µm, respectively) of liver sections stained with hematoxylin and eosin and NAFLD activity score (**B**) or with Oil Red O and its quantification (**C**). Arrows indicate red staining of the lipids droplets. Data are expressed as arbitrary units (a.u.) and presented as mean ± SEM. ns, not significant. Experimental groups: Control mice (C, *n* = 9) and mice exposed to IH followed by reoxygenation (Reox, *n* = 8).

Since there are many confounding factors in patients with OSA that prevent the precise determination of whether CPAP treatment affects liver function and thus the progression of MASLD in these patients, an experimental study was performed. In a previous study, we described an IH protocol for inducing hepatosteatosis in mice [23]. So, to mimic CPAP treatment in patients with OSA, mice exposed to the same protocol followed by 4 weeks of normoxia (reoxygenation, Reox) were used. Control mice were maintained in normoxia (N) throughout the experiment (Figure 2A).

The histological examination of the livers revealed that most of the male mice did not show signs of hepatic steatosis after the reoxygenation period (Figure 2B). Continuing with the study, hepatic lipid content was determined by ORO staining. As indicated in Figure 2C, the intensity of red staining (directly proportional to lipid content) did not show significant differences (*p* = 0.6806) between groups.

Similar results were found in females (Supplementary Figure S1). These data indicate that the normalization of oxygen levels after IH is sufficient to reverse lipid accumulation in the liver of IH mice.

**Figure 3:**
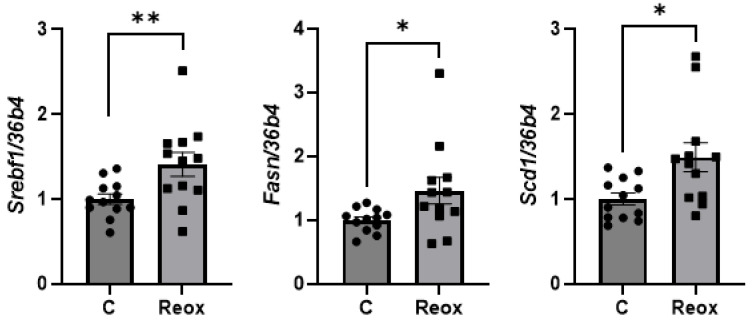
Reoxygenation induces de novo lipogenesis (DNL) in livers of mice exposed to IH. mRNA levels of genes involved in DNL, and normalized to *36b4* expression. Data are expressed as fold increase relative to control condition (C, 1), and presented as mean ± SEM. * *p* < 0.05, ** *p* < 0.01. Experimental groups: Control mice (C, *n* = 12) and mice exposed to IH followed by reoxygenation (Reox, *n* = 12).

Given that the liver is the master organ for lipid homeostasis, we examined the hepatic expression of genes involved in the regulation of lipid metabolism to elucidate the molecular mechanisms underlying the observed effects in the experimental model. As our previous results indicated the upregulation of genes implicated in DNL *(Fasn*, *Scd1*) after IH exposure [24], we first checked the hepatic expression of these genes in IH mice submitted to the reoxygenation protocol. Surprisingly, we found that genes involved in DNL, such as *Srebf1*, *Fasn,* and *Scd1*, are still increased after reoxygenation (Figure 3).

### 3.3. IH Impairs Liver FAO in Mice, Triggering an Increase in Hepatic Oxidative Stress

**Figure 4:**
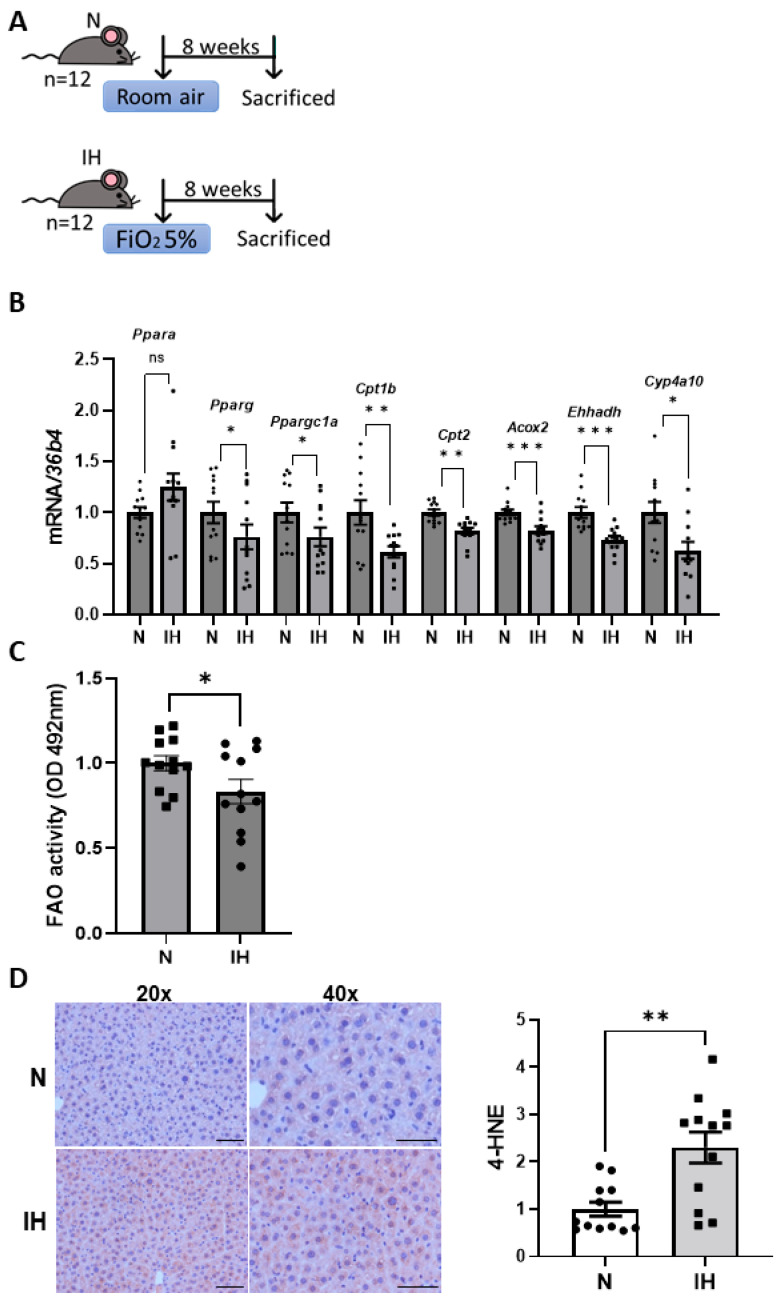
Intermittent hypoxia (IH) impairs hepatic fatty acid oxidation (FAO) and induces oxidative stress in mice. (**A**) Protocol for exposure to IH intervals. IH mice received air with an oxygen fraction (FiO_2_) of 5% in alternating cycles for a total of 8 weeks. Control mice (N) were only exposed to room air. (**B**) mRNA levels of genes involved in FAO normalized to *36B4* gene expression. Data are expressed as fold increase relative to control condition (N, 1) and presented as mean ± SEM. (**C**) Activity of FAO in control and IH mice. Data are expressed as absorbance units and presented as mean ± SEM. (**D**) Immunohistochemistry of 4-HNE and its quantification. Images are shown at 20× and 40× (scale bar at 100 and 50 µm, respectively). Data are expressed as fold increase relative to control condition (N, 1) and presented as mean ± SEM. ns, not significant; * *p* < 0.05, ** *p* < 0.01, and *** *p* < 0.001. Experimental groups: control mice (N, *n* = 12) and mice exposed to intermittent hypoxia (IH, *n* = 12).

Taking into account these results, we evaluated proteins involved in other pathways of lipid metabolism, focusing on FAO. Firstly, we evaluated genes related to FAO in the livers from control and IH mice (Figure 4A) in an attempt to characterize this process in IH conditions, finding that hepatic levels of genes involved in both mitochondrial (*Ppargc1a*, *Pparg*, *Cpt1b*, and *Cpt2*) and peroxisomal β-oxidation (*Acox2*, *Ehhadh*), as well as cytochrome ω-oxidation (*Cyp4a10*), were significantly reduced in IH mice compared to control animals (Figure 4B). Additionally, a functional FAO assay revealed impaired oxidation capacity in livers from mice submitted to IH conditions (Figure 4C), indicating that IH disrupts FAO process.

It is well known that when FAO is impaired, oxidative stress can increase due to the accumulation of lipid intermediates and a disruption in redox balance [25], so next we conducted an immunohistochemistry using an antibody against 4-HNE, a product of lipid peroxidation, in order to quantify oxidative stress levels. The results showed that the livers from IH mice had a higher presence of oxidative stress levels compared to controls (*p* = 0.0016, Figure 4D), as they showed higher 4-HNE signals.

In addition, oxidative stress causes cell damage and lipotoxicity, activating inflammatory pathways in turn. This interplay between oxidative stress and inflammation creates a vicious cycle that favors the progression of MASLD [26]. Taking this into account, we quantified the degree of inflammation, finding that livers from IH mice showed a higher presence of activated macrophages assessed by IBA1 immunostaining, although it was not significant compared to those maintained in normoxic conditions (*p* = 0.0610; Supplementary Figure S2A).

### 3.4. Reoxygenation Induces a Metabolic Reprogramming and Reduces Oxidative Stress in Livers from Mice Exposed to IH

**Figure 5:**
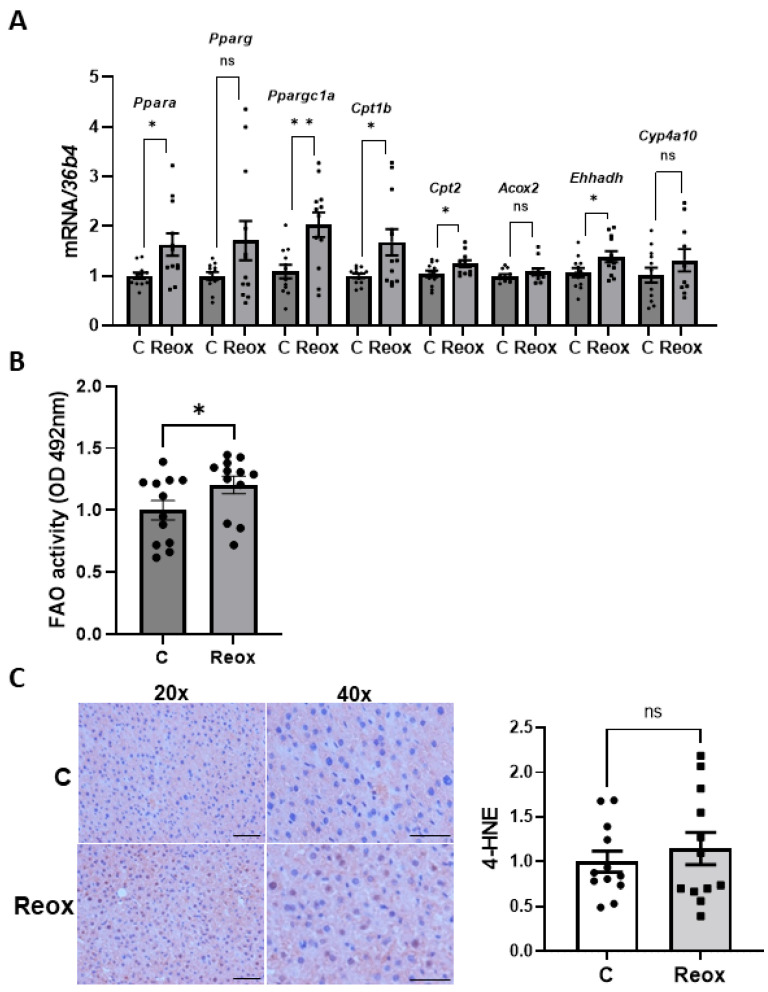
Intermittent hypoxia (IH) induced oxidative stress and inflammation in mouse livers. (**A**) mRNA levels of genes involved in FAO normalized to *36B4* gene expression. Data are expressed as fold increase relative to control condition (C, 1) and presented as mean ± SEM. (**B**) Activity of FAO in control and IH mice. Data are expressed as absorbance units and presented as mean ± SEM. (**C**) Immunohistochemistry of 4-HNE and its quantification. Images are shown at 20× and 40× (scale bar at 100 and 50 µm, respectively). Data are expressed as fold increase relative to control condition (C, 1) and presented as mean ± SEM. ns, not significant; * *p* < 0.05, and ** *p* < 0.01. Experimental groups: Control mice (C, *n* = 12) and mice exposed to IH followed by reoxygenation (Reox, *n* = 12).

When evaluating the effects of reoxygenation, we found that the hepatic content of *Acox2* and *Cyp4a10* was recovered, and the expression levels of *Ppargc1a*, *Ppara, Cpt1b, Cpt2*, and *Ehhadh* were even increased in mice submitted to IH followed by 4 weeks of normoxia (Figure 5A), in parallel to FAO activity (Figure 5B). These results suggest that the normalization of oxygen levels induces a metabolic reprogramming in livers of mice exposed to IH by restoring FAO, which in turn results in a removal of the IH-induced lipid accumulation.

Indeed, after a 4-week reoxygenation period, no differences were found between groups concerning either oxidative stress levels (*p* = 0.3960, Figure 5C) or inflammation (*p* = 0.9494, Supplementary Figure S2B).

### 3.5. CPAP Treatment Normalizes Circulating Levels of β-Hydroxybutyrate, a Marker for Hepatic FAO, in OSA Patients

In order to confirm the results found in the experimental model, we measured the concentration of serum β-hydroxybutyrate (βHB) in the study population since it has been described as a surrogate marker for hepatic FAO [27].

**Figure 6:**
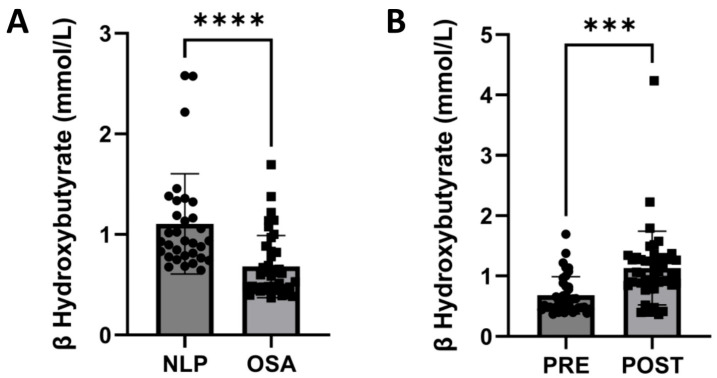
β-hydroxybutyrate levels are impaired in OSA patients and recovered after a CPAP therapy. (**A**) Serum levels of β-hydroxybutyrate in the control (*n* = 32) and OSA patients (*n* = 44), and (**B**) in OSA patients before (PRE) and after (POST) CPAP therapy (*n* = 44). Data are presented as mean ± SD. *** *p* < 0.001, **** *p* < 0.0001.

Interestingly, a significant decrease in βHB serum levels was observed in OSA patients compared with NLP subjects (*p* < 0.0001, Figure 6A). Content of this metabolite was recovered after CPAP treatment (*p* < 0.001, Figure 6B), reflecting that this therapy might improve hepatic FAO impairment in OSA patients.

## 4. Discussion

The present translational research aims to improve the understanding of CPAP treatment by studying the effects of oxygen level normalization (reoxygenation) on metabolic comorbidities associated with OSA, particularly on MASLD onset and/or progression, not only in the clinical setting but also through experimental and molecular approaches in order to achieve a better understanding.

Although no significant differences were found in BMI among the different groups evaluated, the clinical study revealed that OSA is associated with metabolic syndrome comorbidities such as insulin resistance, dyslipidemia, and liver steatosis, according to numerous studies [28,29]. FLI and OWLiver test data revealed a significantly higher prevalence of hepatosteatosis in OSA patients compared to NLP subjects, supporting a previous meta-analysis that indicated a pathogenic link between OSA and MASLD [30].

Although the literature reports inconsistent results on CPAP effectiveness, a 2023 meta-analysis of 31 studies found slight improvements in cholesterol and insulin sensitivity [31]. In line with this, our study found an improved lipid profile, with reduced total cholesterol and VLDL levels in OSA patients after CPAP treatment. Although no statistically significant differences were observed in HOMA-IR when studying the entire population, our study revealed an improvement in the degree of insulin resistance after CPAP in the insulin-resistant OSA group (HOMA-IR ≥ 3.5), suggesting that these patients benefit the most from the metabolic effect of CPAP. However, further studies in larger cohorts are needed to confirm this hypothesis.

Concerning liver function markers, CPAP treatment led to a reduction in transaminase levels, although only AST improvement was significant. This difference may be due to AST not being a liver-specific enzyme, as it is also found in other tissues such as the heart, brain, muscles, and kidneys [32], which may also be affected in OSA, in contrast to ALT, which is the most sensitive indicator of liver cell damage [15]. To date, no studies have reported greater improvement in AST than ALT levels in OSA patients after CPAP therapy, so further research is needed to explore whether the AST improvement has a non-hepatic origin.

Albumin levels, another liver-derived marker, significantly increased after treatment. Although limited, one study supports this finding, reporting similar results in 57 patients after 6 months of CPAP for at least 4 h per night [33]. Altogether, these results might suggest that liver function improves with CPAP, similarly to the meta-analysis that revealed that OSA treatment with CPAP improves liver enzyme levels and reduces hepatic inflammation [34]. Indeed, when studying those patients with a high probability of presenting hepatosteatosis or steatohepatitis at the beginning of the study, defined by both FLI > 60 or OWLiver test, a significant improvement was observed after 18 months of CPAP treatment, suggesting that therapy reduces the probability of MASLD progression. In this regard, it is worth noting that other studies have demonstrated a clear beneficial role of CPAP on MASLD progression, but most were conducted in selected cohorts of patients, such as morbidly obese individuals [35]. On the other hand, Ng et al. conducted a randomized clinical trial with patients with OSA and MASLD, and they found no significant changes in non-invasive markers of MASLD after 6 months of treatment with positive pressure [36], once again, a much shorter CPAP time than in our work, which could explain the discordance between the two studies. Taking into account the scientific evidence available to date, the complex interplay between these conditions needs further research to elucidate underlying mechanisms and optimize intervention strategies.

Thus, we have performed an experimental approach in order to determine whether CPAP treatment affects liver function and MASLD progression. We and others have previously described that IH causes hepatic lipid accumulation in mice, promoting the development of hepatic steatosis [23,37] Here, we have demonstrated that IH also increased the degree of oxidative stress and inflammation in the liver, events previously described in the literature [38,39] Interestingly, the normalization of oxygen levels after IH is sufficient to reverse these effects, highlighting for the first time that reoxygenation can mitigate the deleterious effects induced by IH in the liver. This suggests strong therapeutic potential for treating hypoxia-related liver diseases.

At the molecular level, it has been demonstrated that the hepatosteatosis promoted by IH in mice is accompanied by the upregulation of key genes for hepatic lipid biosynthesis (DNL) such as *Srebp1, Fasn*, and *Scd1* [24,37] In this study, we also analyzed fatty acid oxidation (FAO) genes and found that IH downregulated PPARg expression, in line with previous findings [40], while *Ppara* remained unchanged. Since PPARs are regulators of FAO, it is reasonable that FAO and genes involved in these pathways were also significantly decreased.

Regarding the molecular effects of reoxygenation, we have not found any study in the literature about this issue, conferring high novelty to the results described herein. Beginning with DNL, we observed that lipogenic gene levels remained elevated after reoxygenation. In contrast to the expected result in IH, this cannot be attributed to HIF1α due to prolyl hydroxylases’ activity under normoxic conditions; however, this lipogenic effect might be explained by the upregulation of *Ppara* after the reoxygenation, since it has been demonstrated that this PPAR can also induce hepatic *Srebf1* expression, promoting DNL [41]. Regarding FAO in livers from Reox mice, we observed that the decreased hepatic expression levels of FAO induced by IH were reversed. These results indicate that the normalization of oxygen levels induces a metabolic reprogramming in livers from mice exposed to IH: although DNL is still induced after reoxygenation period, FAO is not impaired; in fact, it is even enhanced, favoring hepatic lipid clearance. Indeed, we presented evidence indicating that CPAP treatment might recover impaired hepatic FAO in OSA patients, as assessed by serum levels of βHB, a metabolite that has been associated with hepatic mitochondrial FAO in MASLD patients [27], among others such as acylcarnitines [42,43].

Altogether, this study revealed, on the one hand, that CPAP improves lipid profiles and hepatic function, decreasing MASLD scores, and on the other hand, that reoxygenation reverses IH-induced hepatic damage and restores fatty acid oxidation pathways. These findings underscore the importance of addressing OSA in the context of metabolic health and MASLD management, while advocating for further research to optimize intervention strategies and proposing CPAP therapeutic potential for addressing both respiratory and metabolic disorders.

## Figures and Tables

**Table 1:** Primer sequences for RT-qPCR.

Gene	Accession Number	Forward (5′ → 3′)	Reverse (5′ → 3′)
m-*36b4*	NM_007475.5	AGATGCAGCAGATCCGCAT	GTTCTTGCCATCAGCACC
m-*Srebf1*	NM_011480.4	GGCCGAGATGTGCGAACT	AGCTGGAGCATGTCTTCGATG
m-*Fasn*	NM_007988.3	CCCTTGATGAAGAGGGATCA	ACTCCACAGGTGGGAACAAG
m-*Scd1*	NM_009127.4	TTCTTGCGATACACTCTGGTGC	CGGGATTGAATGTTCTTGTCGT
m-*Ppara*	NM_011144.6	AGAGCCCCATCTGTCCTCTC	ACTGGTAGTCTGCAAAACCAAA
m-*Pparg*	NM_001308354.2	TCGCTGATGCACTGCCTATG	GAGAGGTCCACAGAGCTGATT
m-*Ppargc1a*	NM_008904.3	AAGTGTGGAACTCTCTGGAACTG	GGGTTATCTTGGTTGGCTTTATG
m-*Cpt1b*	NM_009948.2	CTCCATGGGACTGGTCGATT	CCAAAGTGGCCATACCTTTCC
m-*Cpt2*	NM_009949.3	GAATGACAGCCAGTTCAGGAAGA	CCTTCCCAATGCCGTTCTC
m-*Acox2*	NM_053115.2	TTGATGGAGGCATCCCAAA	CACGTTGGATGAGGCTTTCA
m-*Ehhadh*	NM_023737.3	CCCACTCCCTGGCTATGATC	CAGTTGGACTGATGGCATTGA
m-*Cyp4a10*	NM_010011.3	CACTGGTTCTTTGGGCATGA	TCTCTATGCACGACACAATTTCCT

*36b4*, ribosomal protein lateral stalk subunit P0; *Srebf1*, sterol regulatory element binding transcription factor 1; *Fasn*, fatty acid synthase; *Scd1*, stearoyl-coenzyme A desaturase 1; *Ppara*, peroxisome proliferator activated receptor alpha; *Pparg*, peroxisome proliferator activated receptor gamma; *Ppargc1a*, peroxisome proliferative activated receptor, gamma, coactivator 1 alpha; *Cpt1b*, carnitine palmitoyltransferase 1b; *Cpt2*, carnitine palmitoyltransferase 2; *Acox2*, acyl-coenzyme A oxidase 2; *Ehhadh*, enoyl-coenzyme A, hydratase/3-hydroxyacyl coenzyme A dehydrogenase; *Cyp4a10*, cytochrome P450, family 4, subfamily a, polypeptide 10.

**Table 2:** Clinical variables and non-invasive diagnostic scores of metabolic disorders of the study population.

	Control (N = 32)	OSA (N = 44)	*p*-Value
Age (years)	54.38 ± 8.53	59.36 ± 9.04	**0.0174**
Male sex, *n* (%)	12 (37.5)	30 (68.2)	**0.0079**
Body mass index (kg/m^2^)	28.91 ± 5.54	30.23 ± 6.14	0.3374
AHI (h^−1^)	2.12 ± 1.31	41.11 ± 19.71	**<0.0001**
Glucose (mg/dL)	97.38 ± 13.38	103.40 ± 16.09	0.0902
HbA1c (%)	5.62 ± 0.53	5.71 ± 0.48	0.4435
HOMA-IR	2.93 ± 2.28	4.76 ± 3.52	**0.0120**
Triglycerides (mg/dL)	106.47 ± 66.57	129.80 ± 75.76	**0.0399**
Total cholesterol (mg/dL)	196.81 ± 39.17	188.50 ± 38.21	0.3585
HDL (mg/dL)	57.53 ± 12.96	53.93 ± 19.13	0.0617
LDL (mg/dL)	115.22 ± 30.75	108.30 ± 35.51	0.3780
VLDL (mg/dL)	21.25 ± 13.25	26.20 ± 15.04	**0.0339**
ALT (IU/L)	18.78 ± 7.34	23.52 ± 9.85	**0.0116**
AST (IU/L)	20.28 ± 5.61	22.00 ± 7.21	0.2650
GGT (IU/L)	22.19 ± 13.03	30.20 ± 16.23	**0.0240**
LDH (IU/L)	182.72 ± 36.12	182.20 ± 34.82	0.9478
ALP (IU/L)	69.63 ± 22.69	68.05 ± 20.64	0.7530
Total bilirubin (IU/L)	0.57 ± 0.48	0.57 ± 0.32	0.340
Albumin (g/dL)	4.38 ± 0.27	4.41 ± 0.25	0.5677
Platelets (10^9^/L)	226.31 ± 71.27	234.10 ± 51.73	0.5828
Iron (µg/dL)	78.47 ± 33.50	87.66 ± 33.22	0.2391
C-Reactive Protein (mg/L)	0.42 ± 0.59	0.31 ± 0.24	0.9979
FLI	46.33 ± 28.65	66.26 ± 29.25	**0.0042**
NAFLD FS	−1.43 ± 1.29	−1.14 ± 1.03	0.2706
HFS	0.06 ± 0.07	0.07 ± 0.10	0.4614

Data are shown as mean ± SD or number of cases (%), analyzed using unpaired test for quantitative variables in control vs. OSA analysis: unpaired *t*-test for parametric variables or Mann-Whitney test for non-parametric variables. For categorical variables, data were analyzed using Chi-Square Test or Fisher’s exact test (depending on the number of patients) in controls vs. OSA patients. OSA, obstructive sleep apnea; AHI, apnea-hypopnea index; HbA1c, glycated hemoglobin; HOMA-IR, Homeostatic Model Assessment-Insulin Resistance; HDL, high-density lipoprotein cholesterol; LDL, low-density lipoprotein cholesterol; VLDL, very low-density lipoprotein cholesterol; ALT, alanine aminotransferase; AST, aspartate aminotransferase; GGT, gamma-glutamyl transferase; LDH, lactate dehydrogenase; ALP, alkaline phosphatase; FLI, Fatty Liver Index; NAFLD FS, Non-Alcoholic Fatty Liver Disease Fibrosis Score; HFSt, Hepamet Fibrosis Score.

**Table 3:** Clinical variables and non-invasive diagnostic scores of metabolic disorders of OSA population pre- and post-treatment.

Features	OSA (N = 44)		*p*-Value
	Pre-CPAP (N = 44)	Post-CPAP (N = 44)	
Age (years)	59.36 ± 9.04	61.20 ± 9.19	**<0.0001**
Male sex, *n* (%)	30 (68.2)	30 (68.2)	1.0000
Body mass index (kg/m^2^)	30.23 ± 6.14	30.12 ± 5.25	0.7755
AHI (h^−1^)	41.11 ± 19.71	7.12 ± 15.73	**<0.0001**
Glucose (mg/dL)	103.40 ± 16.09	105.30 ± 18.40	0.3246
HbA1c (%)	5.71 ± 0.48	5.64 ± 0.47	0.1613
HOMA-IR	4.76 ± 3.52	4.86 ± 4.08	0.7838
Triglycerides (mg/dL)	129.80 ± 75.76	112.20 ± 56.74	0.0656
Total cholesterol (mg/dL)	188.50 ± 38.21	175.30 ± 36.08	**0.0044**
HDL (mg/dL)	53.93 ± 19.13	53.64 ± 13.16	0.0663
LDL (mg/dL)	108.30 ± 35.51	97.66 ± 34.61	0.0799
VLDL (mg/dL)	26.20 ± 15.04	22.45 ± 11.31	**0.0304**
ALT (IU/L)	23.52 ± 9.85	23.11 ± 10.27	0.3311
AST (IU/L)	22.00 ± 7.21	19.68 ± 6.35	**0.0088**
GGT (IU/L)	30.20 ± 16.23	29.84 ± 16.86	0.6819
LDH (IU/L)	182.20 ± 34.82	169.40 ± 34.55	**0.0002**
ALP (IU/L)	68.05 ± 20.64	74.93 ± 24.97	**0.0011**
Total bilirubin (IU/L)	0.57 ± 0.32	0.57 ± 0.29	0.8502
Albumin (g/dL)	4.41 ± 0.25	4.61 ± 0.21	**<0.0001**
Platelets (109/L)	234.10 ± 51.73	230.30 ± 52.06	0.4296
Iron (µg/dL)	87.66 ± 33.22	93.73 ± 29.10	0.2265
C-Reactive Protein (mg/L)	0.31 ± 0.24	0.24 ± 0.19	0.0599
FLI	66.26 ± 29.25	64.62 ± 28.14	0.3828
NAFLD FS	−1.14 ± 1.03	−1.22 ± 1.20	0.4821
Hepamet FS	0.07 ± 0.10	0.07 ± 0.09	0.7298

Data are shown as mean ± SD or number of cases (%), analyzed using paired test in pre- and post-CPAP: paired *t*-test for parametric variables or Wilcoxon test for non-parametric variables. For categorical variables, data were analyzed using McNemar’s test. OSA, obstructive sleep apnea; CPAP, continuous positive airway pressure; AHI, apnea-hypopnea index; HbA1c, glycated hemoglobin; HOMA-IR, Homeostatic Model Assessment—Insulin Resistance; HDL, high-density lipoprotein cholesterol; LDL, low-density lipoprotein cholesterol; VLDL, very low-density lipoprotein cholesterol; ALT, alanine aminotransferase; AST, aspartate aminotransferase; GGT, gamma-glutamyl transferase; LDH, lactate dehydrogenase; ALP, alkaline phosphatase; FLI, Fatty Liver Index; NAFLD FS, Non-Alcoholic Fatty Liver Disease Fibrosis Score; Hepamet, Hepamet Fibrosis Score.

## Data Availability

The authors declare that all data supporting the findings of this study are available within the paper. The raw data that support the findings of this study are available from the corresponding author upon reasonable request.

## Supplementary Materials

The following supporting information can be downloaded at https://www.mdpi.com/article/10.3390/antiox14080971/s1, Figure S1: Reoxygenation is sufficient to reverse the hepatic lipid accumulation induced by intermittent hypoxia (IH) in female mice.; Figure S2: Reoxygenation reduces IH-induced inflammation in mice.
