# Supplementary material for: Normalization of Oxygen Levels Induces a Metabolic Reprogramming in Livers Exposed to Intermittent Hypoxia Mimicking Obstructive Sleep Apnea
Source: Antioxidants (Basel). 2025 Aug 7;14(8):971. doi: 10.3390/antiox14080971 (PMC12383098; doi:10.3390/antiox14080971)
Supplement: Supplementary file 1 [file antioxidants-14-00971-s001.zip › antioxidants-3704555-supplementary.pdf]

Supplementary Figure S1

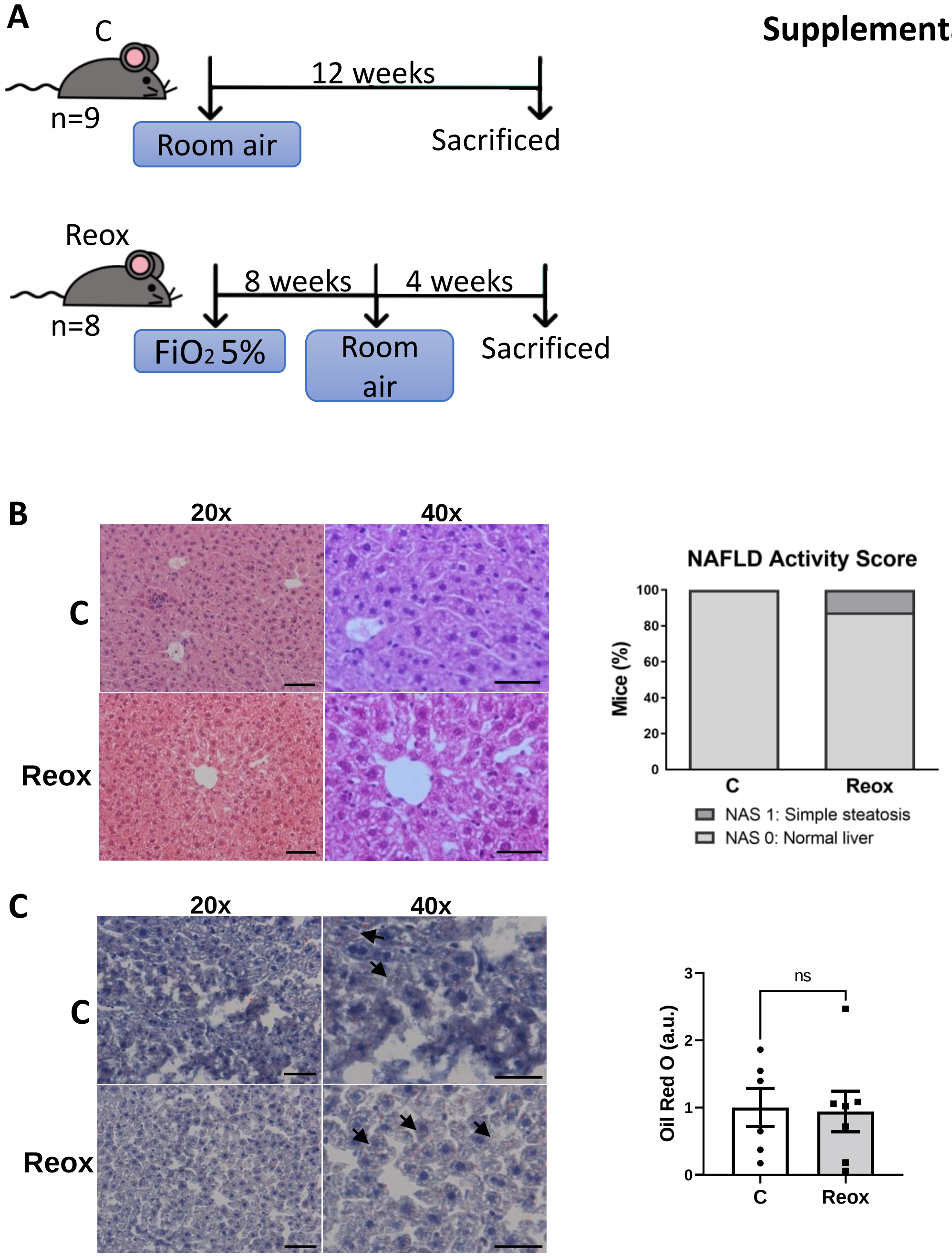

Supplementary Figure S1. Reoxygenation is sufficient to reverse the hepatic lipid accumulation induced by intermittent hypoxia (IH) in female mice. (**A**) Protocol for exposure to IH intervals followed by reoxygenation (Reox). Reox mice received air with an oxygen fraction (FiO_2) of 5% in alternating cycles for a total of 8 weeks, followed by 4 weeks of room air exposure. Control mice (N) were only exposed to room air. (**B**,**C**) Representative images at 20× and 40× (scale bar at 100 and 50μm, respectively) of liver sections stained with Hematoxylin and Eosin and NAFLD activity score (**B**) or with Oil Red O and its quantification (**C**). Arrows indicate red staining of the lipids droplets. Data are expressed as arbitrary units (a.u.) and presented as mean ± SEM. ns, not significant. Experimental groups: control mice (C, n = 6) and mice exposed to IH followed by reoxygenation (Reox, n = 7).

Supplementary Figure S2

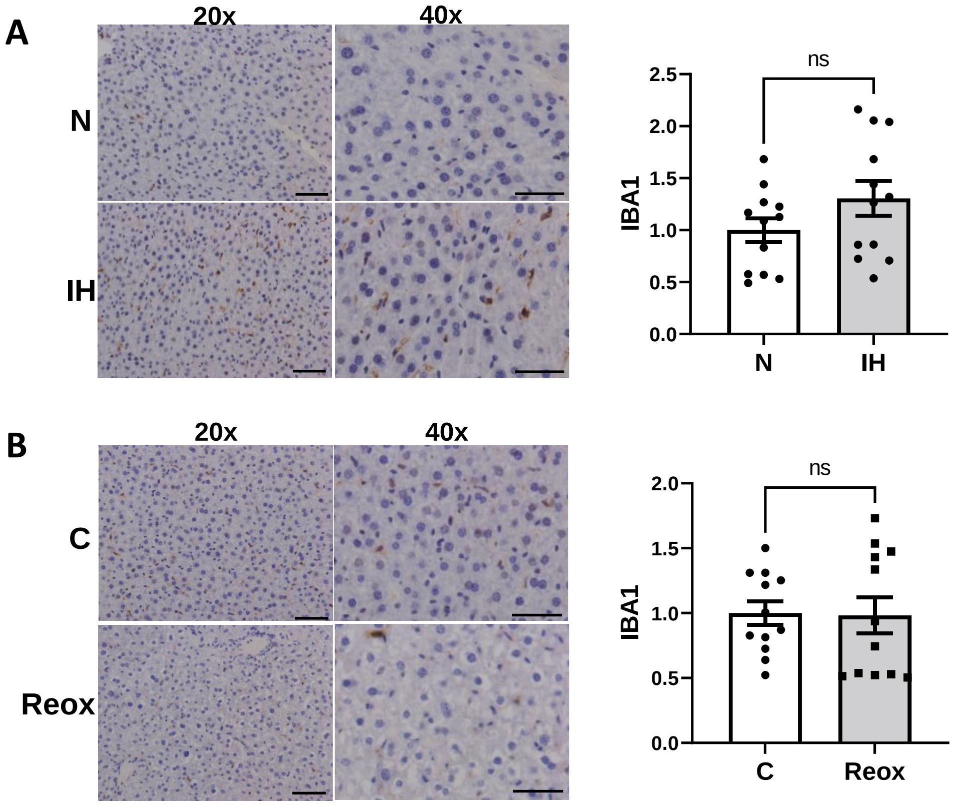

Supplementary Figure S2. Reoxygenation reduces IH-induced inflammation in mice. Immunohistochemistry of IBA1 and its quantification. Images are shown at 20× and 40× (scale bar at 100 and 50µm, respectively). Data are expressed as fold increase relative to control condition (1) and presented as mean ± SEM. ns, not significant. Experimental groups: (**A**) Control mice (N, n=12) and mice exposed to intermittent hypoxia (IH, n=12). (**B**) Control mice (C, n=12) and mice exposed to IH followed by reoxygenation (Reox, n=12).
